# Development of alkali activated paver blocks for medium traffic conditions using industrial wastes and prediction of compressive strength using random forest algorithm

**DOI:** 10.1038/s41598-023-42318-4

**Published:** 2023-09-13

**Authors:** A. Chithambar Ganesh, R. Mohana, Parthiban Loganathan, Vinod M. Kumar, Mehmet Serkan Kırgız, N. Nagaprasad, Krishnaraj Ramaswamy

**Affiliations:** 1 Department of Civil Engineering, Vel Tech Rangarajan Dr. Sagunthala R&D Institute of Science and Technology, Chennai, India; 2 Department of Civil Engineering, Mepco Schlenk Engineering College, Sivakasi, India; 3 Department of Civil Engineering, Sree Vidyanikethan Engineering College, Chennai, India; 4 Northwestern University, Chicago, IL 60208-201 USA; 5 Department of Mechanical Engineering, ULTRA College of Engineering and Technology, Madurai, Tamil Nadu 625104 India; 6 Centre for Excellence-Indigenous Knowledge, Innovative Technology Transfer and Entrepreneurship, Dambi Dollo University, Dembi Dolo, Ethiopia; 7 Department of Mechanical Engineering, College of Engineering and Technology, Dambi Dollo University, Dembi Dolo, Ethiopia

**Keywords:** Chemistry, Energy science and technology, Engineering, Materials science, Physics

## Abstract

Geopolymer is an environment friendly construction material that could be synthesized using either the natural source or the industrial byproducts such as flyash and GGBS. The characteristics of the Geopolymer rely on the proportion of the flyash and GGBS and the concentration of the activator solution used. In this research work, the effect of partial replacement of flyash with GGBS in proportions such as 0, 10, 20, 30 and 40% is investigated. Also Molarity of NaOH are tested from 8 to 14 M and both the parameters are optimized. In this optimized Geopolymer concrete, the utilization of iron slag as a partial substitute for river sand in various proportions such as 10, 15, 20, 25, 30 35, 40 and 45% are investigated. The optimized Geopolymer concrete with iron slag is investigated for its performance as a paver block with incorporation of banana fiber in proportions such as 0, 0.5, 1 and 1.5 and is compared with conventional cement concrete paver block. The results show that there is a significant enhancement in the properties of Geopolymer concrete with the different levels of optimization and the utilization of natural banana fiber. The developed sustainable paver block was found to with stand medium traffic conditions as per IS 15658:2006. Further this study employed random forest (RF) algorithm for the prediction of compressive strength of geopolymer concrete specimens for the variable parameters such as molarity of alkaline solution, Flyash/GGBS ratio and partial replacement of river sand with iron slag. The performance evaluation parameters represented high accuracy of developed RF model. This research work unleashes a heft potential of Geopolymer concrete to develop economical eco-friendly sustainable paver blocks to the society through mitigation of environmental strain on the ecosystem.

## Introduction

In the recent decade, significant research works have been concentrated in the vicinity of Geopolymer as a construction material due to its highest versatility. Owing to its outstanding property of being able to synthesise concrete without cement content, geopolymer concrete (GC) gains importance as an eco-friendly and sustainable building material as it addresses the acute environmental issues caused by the manufacture of cement. Geopolymer is inorganic and is synthesized by the activation of alumina silicate base compounds using alkaline solution^1–3^. The base materials are available as a natural source such as kaolinite, metakaolin or industrial effluents such as Ground Granulated Blast Furnace Slag (GGBS), fly ash, high calcium wood ash, rise husk ash, waste glass powder^4–10^. Duxson et al.^11^ reported early strength attainment with the slag-based geopolymer matrix and enhanced durability with the fly ash-based geopolymer concrete (FGC). FGC concrete requires heat curing for the effective gaining of strength. Yildirim and Prezzi performed various tests and analyzed the suitability of unused slag for the use as construction material and reported superior performance than the conventional materials in the field. Sithole et al., stated the ability of GGBS to produce a sustainable concrete with the inclusion of foundary sand as the fine aggregate instead of natural sand.

Deb et al.^12^ utilized GGBS as a partial substitute for FGC and stated the possibility of synthesis of Geopolymer concrete under ambient curing. Investigational works performed by Hamidi et al.^13^ and Hardjito et al.^14^ report that the molarity of the alkaline solution and curing condition hold a major part in the strength-gaining process of GC. The binding potency of GC is primarily affected due to the concentration of the alkaline solution and the category of base materials involved in the process. Hence effort can be concentrated towards the synthesis of Geopolymer concrete with optimum fly ash–slag blend ratio and optimum concentration of an alkaline solution^12,15,16^.

Many research findings document high compressive properties and increased resistance against acid and fire attacks with low creep in Geopolymer concrete^17–26^. At high temperatures, geopolymer concrete performs better than cement concrete with increased retained compressive strength. This is due to the sturdy bond that exists within the steel and matrix than the cement concrete^27,28^. In addition, geopolymer concrete proves to be cost-beneficial when compared to conventional concrete with a reduced economic index^29^. The infirmity of Geopolymer is its brittle character. The fracture of Geopolymer was observed to be smoother than the conventional concrete^30^. Pan et al.^31^ stated that the brittleness is because of the morphology of the binder material that alters the binder inside the matrix. Contemporary advancement in the concrete industry is fibre-reinforced concrete. The existence of fibres improves the structural integrity and reduces brittleness^32,33^. Many research work report that the integration of fibre augments the hardened properties of the concrete^34–37^. Additions of synthetic fibres reduce the brittleness and improve the impact energy^38–40^. Considering the economic importance, natural fibres can be employed to reduce the brittleness of the GC. Out of many available natural fibres, owing to the high tensile strength and young’s modulus, Banana fibres are preferred to coconut or sugarcane bagasse fibre^41–44^. Hence by incorporating banana fibres, an endeavour can be focused towards reducing this brittleness of geopolymer concrete.

Geopolymer is used in the production of bricks, boat ramps, panels for walls, water tanks and paver blocks^45,46^. Geopolymer paver blocks synthesized in various research works were found to perform well only under low traffic conditions as prescribed by IS 15658: 2006^47^. Analysis of failed paver block claims the low impact energy and brittleness of the Geopolymer concrete as the reason for failure. Hence an approach can be made in the production of a Geopolymer paver block to reduce its brittleness and match the medium traffic conditions as prescribed by IS 15658: 2006^47^.

Gavriletea et al.^48^ reported a global imbalance in the supply and demand of the sand market with the major share being held by the construction industry. Another problem faced by the construction sector is the scarcity of river sand. Adding to this point, river sand mining leads to serious environmental problems. Padmalal et al.^49^ conducted a field survey and claimed that river sand mining accounts for bank erosion, affected surface water and aquatic flora and fauna, the social life of the locality and lowered groundwater. Sand mining leads to the undermining of spillways, abutments and piers. Anthony et al.^50^ claimed sand mining as an important reason for the reduced supply of sediments to the coast and bank erosion. The intrusion of seawater was observed due to sand mining which affected the cultivation of the locality. Many research works recommended identifying a new material to replace the river sand as fine aggregate^50–52^. On the other hand, there is a problem with the disposal of iron slag, a waste residue that comes out of the iron industry. Noufal and Singh^53^ and Singh and Siddique^54^ reported the enhancement of mechanical characteristics of conventional cement concrete by replacing river sand with iron slag. Hence an endeavour can be made to replace the river sand with iron slag inside the optimized geopolymer concrete.

Many research works concentrate on the development of GC. But only a few focus towards the optimization of different factors that alter the geopolymerization and proceeding with the product development. This research work focuses the limelight on the production of sustainable eco-friendly paver blocks using various industrial wastes like fly ash, GGBS, iron slag and banana fibres. The novelty of this research study is the optimization of important factors for geopolymerization, utilization of iron slag in optimized GC and further optimization of incorporation of natural fibres in optimized Geopolymer concrete. Further, an analytical study using the random forest algorithm is carried out to develop a relationship between the output compressive strength and input variables such as concentration of activator solution, fly ash/GGBS ratio and fractional substitute of river sand with iron slag. The developed model for the prediction of compressive strength is validated using the evaluation parameters. The research hypothesis is to develop a sustainable eco-friendly economical paver block using industrial waste materials such as fly ash, GGBS and iron slag.

## Materials and methodology

### Geopolymer matrix

**Figure 1:**
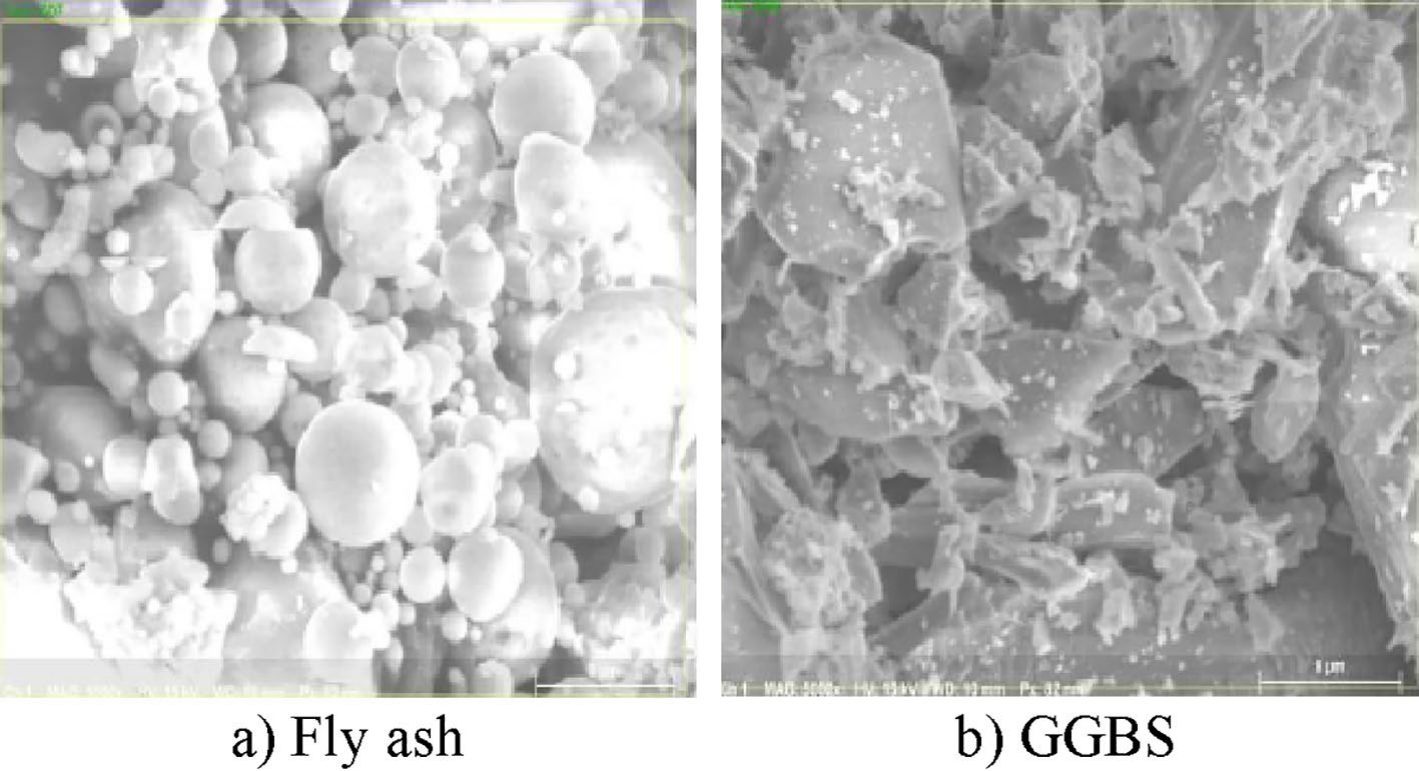
SEM analysis.

The preliminary resources used to synthesise geopolymer binder in this research work are binder base materials, fly ash, GGBS, and alkaline solution such as NaOH solution and Na_2_SiO_3_ solution. Low calcium Class F type, collected from Tuticorin thermal power station and High calcium GGBS procured from Salem steel plant, is utilized in this study. SEM view of fly ash and GGBS is depicted in Fig. 1.

Specific gravity (SG) of fly ash and GGBS is found as 2.3 and 2.92. The chemical compositions of fly ash and GGBS utilized are listed in Table 1.

**Table 1:** Chemical composition of materials used.

Chemical Composition	Fly ash	GGBS	Iron Slag
Al_2_O_3_	17.71	14.42	0
Fe_2_O_3_	1.21	1.11	16.11
SiO_2_	77.10	37.73	44.67
CaO	0.62	37.34	5.52
MgO	0.90	8.71	1.75

A concoction of NaOH solution and Na_2_SiO_3_ solution is used as the alkaline solution. Sodium silicate (Na_2_SiO_3_) is a commonly used precursor in the synthesis of geopolymer materials. While it is true that some studies have shown that NaOH alone can be used for geopolymerization, Sithole and Mashifana compared the activation of GGBS with KOH and NaOH and claimed the better performance of Sodium based activator than the potassium based activator. Sithole et al. used different concenterations of NaOH and suggested that NaOH is sufficient for the activation of GGBS based geopolymer concrete. But in this study, a blend of flyash and GGBS is performed, hence combination of NaOH and Na_2_SiO_3_ is preferred. The use of sodium silicate as a precursor can have several advantages. sodium silicate is a source of both sodium and silica, which are the two main components required for the formation of geopolymer materials. sodium silicate can help to control the setting and hardening of the geopolymer material. The addition of sodium silicate can lead to the formation of a gel-like precursor, which can be important for controlling the reaction kinetics and ensuring the final properties of the material. NaOH flakes and Na2SiO3 solution are brought as such from chemical industries. SG of NaOH and Na_2_SiO_3_ is 1.47 and 1.6. Locally available gravel stones of 10 mm size are used as the coarse aggregate (CA), and locally existing granular river sand is utilized as the fine aggregate. SG and fineness modulus of fine aggregate is 2.62 and 2.3 as per IS: 383-1970^55-57^. River sand confirms to Zone-2. SG and fineness modulus of CA is 2.97 and 6.95 as per IS: 383-1970.

### Cement

Conventional cement concrete control specimen is made using Ordinary Portland Cement (OPC) of grade53 that confirms IS: 12269-2013^58^. SG of cement is determined to be 3.14. The initial and final setting times are determined as 30 and 513 min.

### Super plasticizer

A Superplasticizer of polycarboxylic ether formulation type, MasterGlenium SKY 8233, is utilized in this research work. This is a high-range water reducer and is applied to boost the workability of the GC significantly. SG of admixture is given as 1.08 by the manufacturer, and the properties are in accordance with ASTM C494^59^. Application of this plasticizer ensures well-dispersed particle suspension and reduces the risk of bleeding and segregation. Admixture is added during the wet state after the addition of about 60% of the alkaline solution.

### Banana fiber

Natural fibres are preferred over the utilization of synthetic fibres for economically sustainable development. Owning to the high tensile strength and young’s modulus, Banana fibres are utilized in the present investigation with the objective of increasing the energy absorption capacity of the paver block. Banana fibres used are obtained from the pseudo stem of a banana tree locally available. The properties of banana fibres are tabulated in Table 2. The plant we have used in this report was collected from some local market in Chennai, Tamilnadu, India. This study complies with relevant legislation and international, national, and institutional guidelines.

**Table 2:** Properties of banana fibre.

Fiber	Diameter (mm)	Length (mm)	Aspect Ratio	Tensile Strength (MPa)	Young’s Modulus (GPa)	Elongation (%)	Density (kg/m)
Banana Fiber	0.15	6	40	581	28–50	5.3	780

### Iron slag

Iron slag, a byproduct of the pig iron industry obtained from the Kamachi Iron and Steel industry situated at Gummidipoondi, is utilized in this research work. Iron slag is black–grey in colour and is lighter and more brittle than the river sand. Initially, these iron slags were subjected to screening through the 4.75 mm sieve, and all the oversized particles were removed. SG and FM of Iron Slag Waste are determined as 2.48 and 2.4 as per IS: 383-1970^57^. Irons slag confirms Zone I grading. The chemical concentrations of the iron slag are listed in Table 1.

### Experimental methodology

The experimental investigation aims at the development of sustainable high-impact strength paver blocks. The extensive methodology involves the optimization of important parameters that affects geopolymerization, such as the molarity of the alkaline solution and fly ash-GGBS blend ratio. The optimum utilization of iron slag as a fractional substitute of river sand as fine aggregate is investigated over the optimized geopolymer mortar samples. Finally, this optimized geopolymer matrix, along with the iron slag, is subjected to the application-oriented study in the form of a paver block with fibres and is compared with the conventional cement paver blocks for its mechanical properties and water absorption capacity.

In the first phase, the optimization of the molarity of the NaOH solution that is utilized as the alkaline solution in various proportions, such as 8, 10, 12, and 14 M, is determined. The geopolymer mortar specimens with the optimized molarity are then investigated for the effective utilization of fly ash and slag as alumina silicate source material in various proportions such as 100/0, 90/10, 80/20, 70/30 and 60/40. These optimized Geopolymer specimens are then investigated for the effectual consumption of iron slag as a fractional substitute for river sand in various fractions such as 0, 10, 15, 20, 25, 30, 35, 40 and 45% to determine the effective utilization of iron slag inside geopolymer matrix. Finally, this optimized geopolymer mix is applied as a paver block of dimensions 200 × 100 × 60 using various proportions of natural banana fibres such as 0.5, 1 and 1.5 percent, and its compressive, tensile, flexural and impact strength and water absorption capacity are contrasted with the conventional cement-based paver blocks. Further SEM analysis is carried out over the optimized specimen to understand the microstructure of the matrix.

### Mix design, specimen specification, and optimization of geopolymer

Geopolymer specimens are designed for M35 grade using B.V. Rangan mix design^60^ in accordance with IS 10262:2009^61^. To achieve high strength Interfacial Transition Zone, a high solution-to-binder ratio of 0.61 is fixed. The ratio of Na_2_SiO_3_ to NaOH is set as 2.5. The molarity of the NaOH solution and the combined utilization of fly ash-GGBS slag is optimized in the present study.

An alkaline solution is made ready 24 h before the day of casting, and all the proportioned ingredients are assorted using a pan mixer for about 300 s. Geopolymer concrete is then subjected to a compaction factor test to identify the workability of the matrix. The compaction factor test procedure is as follows; (1) the sample of concrete to be tested shall be placed gently in the upper hopper, using the hand scoop, (2) the hopper shall be filled level with its brim, and the trap door shall be opened so that the concrete falls into the lower hopper, (3) certain mixes have a tendency to stick in one or both of the hoppers. If this occurs, the concrete may be helped by pushing the rod gently into the concrete from the top, (4) immediately after the concrete has come to rest, the cylinder shall be uncovered, the trap door of the lower hopper opened, and the concrete allowed to fall into the cylinder, (5) the excess concrete remaining above the level of the top of the cylinder shall then be cut off by holding a trowel, (6) weight the cylinder with concrete to the nearest 10 g. This weight is known as the weight of partially compacted concrete (W1), (7) empty the cylinder and then refill it with the same concrete mix in layers approximately 5 cm deep, each layer being heavily rammed to obtain full compaction, (8) level the top surface, (9) weigh the cylinder fully compacted. This weight is known as the weight of fully compacted concrete (W2), (10) weight of empty cylinder + hand compacted concrete, which is known as W3, (11) Find the weight of the empty cylinder (W). And it was calculated with W_2_-W_1_/W_3_-W_1_. The quantity of the compaction factor is evaluated as follows. If the factor is lower than 0.78, 0.85, 0.92, and 0.95, the fresh state of a material are known as very low quality, low quality, medium quality, and high quality, respectively.

Concrete is then cast into moulds for testing. All the geopolymer specimens are immediately subjected to heat curing at 75 degrees Celsius in a hot air oven for 12 h. The specimens are then kept at ambient temperature, and moulds are removed after 1 day. Mix proportions for the different specimens are listed in Table 3. To evaluate the performance of geopolymer paver block specimens, cement concrete paver block specimen is made of conventional cement as per IS 10262:2009 in the ratio of 1:1.72:2.2.

**Table 3:** Mix proportions of specimen type.

Specimen Type	Fly ash (kg/m^3^)	GGBS (kg/m^3^)	Molarity of Alkali (M)	CA (kg/m^3^)	River sand (kg/m^3^)	Iron Slag (kg/m^3^)	Banana Fiber (%)
G8	610	0	8	872.57	452.07	0	0
G10	610	0	10	872.57	452.07	0	0
G12	610	0	12	872.57	452.07	0	0
G14	610	0	14	872.57	452.07	0	0
G12G10	549	61	12	883.11	457.32	0	0
G12G20	488	122	12	893.65	462.99	0	0
G12G30	427	183	12	904.19	468.45	0	0
G12G40	366	244	12	914.73	473.90	0	0
OGI10	488	122	12	893.65	425.76	44.78	0
OGI15	488	122	12	893.65	402.10	67.17	0
OGI20	488	122	12	893.65	378.45	89.56	0
OGI25	488	122	12	893.65	354.80	111.95	0
OGI30	488	122	12	893.65	331.15	134.33	0
OGI35	488	122	12	893.65	307.49	156.73	0
OGI40	488	122	12	893.65	283.84	179.12	0
OGI45	488	122	12	893.65	260.19	209.51	0
F0.5OGI35	488	122	12	893.65	307.49	156.73	0.5
F1OGI35	488	122	12	893.65	307.49	156.73	1
F1.5OGI35	488	122	12	893.65	307.49	156.73	1.5

## Results and discussion

### Workability of GC

The feasibility of synthesis GC with optimum molarity, fly ash-GGBS slag blend ratio and iron slag content is investigated by studying the workability of the specimens. The compaction factor of the GC specimens is determined using the Compaction factor test as per IS1199-2013^62^. The findings are listed in Table 4.

**Table 4:** Compaction factor of geopolymer specimens.

Specimen Type	Compaction factor	Degree of workability
G8	0.91	Medium
G10	0.91	Medium
G12	0.90	Medium
G14	0.89	Medium
G12G10	0.89	Medium
G12G20	0.88	Medium
G12G30	0.87	Low
G12G40	0.86	Low
OGI10	0.87	Low
OGI15	0.87	Low
OGI20	0.88	Medium
OGI25	0.88	Medium
OGI30	0.87	Low
OGI35	0.86	Low
OGI40	0.85	Low
OGI45	0.85	Low
F0.5OGI35	0.84	Low
F1OGI35	0.83	Low
F1.5OGI35	0.80	Very Low

It is pragmatic from Table 4 that the workability of the GPC specimens decreases with the rise in the molarity of the NaOH solution. This decrease could be attributed due to the rationale that with the increase in molarity, the concentration of the alkaline solution increases, it increases the concentration of a solution, increasing the viscosity, thereby affecting the workability of the concrete. It is inferred that the workability of the specimen with the highest molarity is medium, as per British Road Note 4 Standards^63^.

From the compaction factor results, it is observed that the incorporation of GGBS decreases the workability of GC. The reduction of compaction factor is ascribed to the higher specific gravity and specific surface area, texture and irregular shape of GGBS. Also, the CaO content in GGBS gets liquefied immediately in the process of polymer formation resulting in a stiffer matrix. Research works by Deb et al.^14^ and Hassan et al.^64^ also confirm the same.

From Table 4, it is inferred that initially, there is an augment in the workability of the geopolymer specimens with the utilization of iron slag till 25%; beyond that, there is a reduction in the workability to 45 percent. The initial increase in workability is because of the less specific gravity and fine granular structure of the iron slag. Singh and Siddique^54^ reported the high water-absorbing capacity of iron slag. Hence the decrease in workability at the increased dosage of iron slag could be attributed to the change in its property caused due to its water-absorbing property. According to British Road Note 4 Standards, from Table 4, it is inferred that the workability of GC specimens with optimum molarity, optimum fly ash and GGBS content and optimum iron slag content is low with and without the fiber content. This demands the need for the application of machine compaction for better structural integrity of the matrix.

### Compressive strength (CS) of GC

The optimization of primary factors of geopolymerization, such as the molarity of the alkaline solution and fly ash-GGBS blend ratio, is investigated through the assessment of the compressive strength of the GC. The influence of utilization of iron slag as a fractional substitute for river sand is also investigated. CS of the various casted GC samples for the different mix constituents, as listed in Table 3, are determined in accordance with IS 516-2021^65^ using a CTM of 2000kN capacity. The outputs are listed in Table 5.

**Table 5:** Compressive strength of geopolymer specimens.

Specimen Type	Compressive Strength (MPa)	
	7 Days	28 Days
G8	33.4	38.4
G10	35.0	40.2
G12	36.7	42.2
G14	35.6	41.3
G12G10	37.5	42.6
G12G20	40.0	44.9
G12G30	39.7	44.3
G12G40	38.6	42.4
OGI10	40.2	45.2
OGI15	40.4	45.4
OGI20	40.6	46.0
OGI25	41.5	46.6
OGI30	42.0	47.1
OGI35	43.2	48.5
OGI40	42.8	48.0
OGI45	42.6	47.8

**Figure 2:**
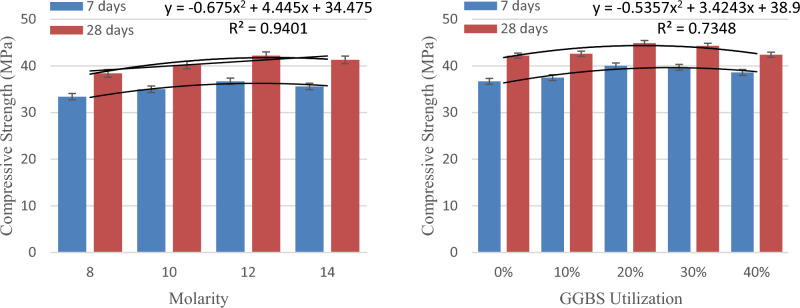
Variation of compressive strength with molarity and GGBS utilization.

The relationship between the fly ash-GGBS blend and the molarity of the NaOH solution with the CS is depicted in Fig. 2.

Figure 2 reports an enhancement in CS of the GC as the molarity of the solution increases, and G12 specimens with 12 M yield a maximum CS than the other specimens. Increased dissolubility of Al-Si ions that exist in the fly ash due to the existence of an alkaline solution leads to improved geopolymerization, which adds up the strength. Chindaprasirt et al.^66^reported enhancement in CS are because of the enhanced leaching of binder base materials by the activator solution in the flash-based GC.G12 specimens with 12 M gained 86% of the 28 days strength in the first seven days itself due to heat curing.

From Fig. 2, it is explicit that Geopolymer specimen with 80%flyash and 20% GGBS shows maximum compressive strength than the other specimens. Initially, with the 20% incorporation of GGBS, the compressive strength increases initially, but the strength decreases with the 40% utilization of GGBS. The initial augmentation in CS corresponds to the existence of CaO in GGBS. The reduction in compressive strength beyond 20% is perceived during the mixing and the conductance of the workability test. G12G40 specimens are stiffer than G12G20 specimens, while the production itself is due to the presence of more GGBS content. This stiffer matrix, when exposed to heat curing, immediately becomes drier and affects the matrix homogeneity, which paves the way for the decline in the compressive strength. The optimum G12G20 specimens gained 89% of the 28 days strength within 7 days due to heat curing^66,67^.

**Figure 3:**
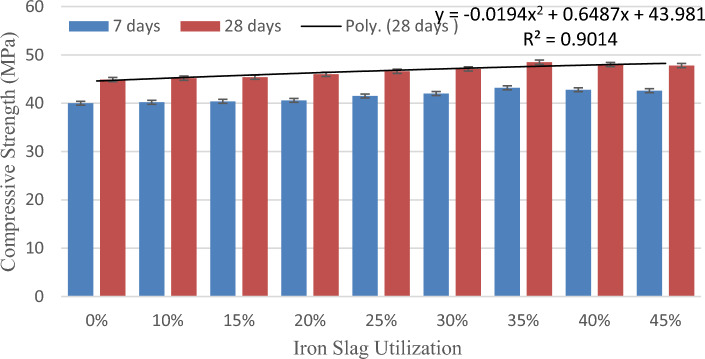
Variation of compressive strength with iron slag.

Figure 3 depicts increase in compressive strength till 35 percent of fractional substitution of river sand with iron slag; beyond that, it decreases. This embarks the optimum % of substitution with iron slag inside geopolymer concrete as 35 percent with 8 percent increase in compressive strength of fly ash-GGBS blend GC.

The augment in strength till the optimum percent is due to the presence of Ca and silicates that reacts with the alumina elements in fly ash and GGBS leading to the enhancement of the compressive strength. Silicates present in the iron slag involve actively in the geopolymerization reaction. Alzaed^63^ studied the outcome of utilizing iron fillings in cement concrete and claimed a raise in compressive strength of about 17% with 30 percent utilization of iron slag. Singh and Siddique^54^ reported increase of about 20 percent in CS with the employment of iron slag in self- compacting concrete.

From the above discussions, the following factors are optimized:

- Molarity of the sodium hydroxide solution as 12 M,
- 80/20 fly ash/GGBS ratio as the binder source material,
- 35% of River sand was partially replaced by iron slag.

### Performance of paver blocks

GC specimens with the optimized molarity, binder source material and iron slag as the filler material are cast as paver blocks of dimension200 × 100 × 60 mm with different proportions of natural banana fibers such as 0.5, 1 and 1.5 percent, and its compressive, tensile, flexural and impact strength and water absorption capacity are determined and contrasted with the cement-based paver blocks.

#### CS of paver blocks

The performance of the optimized geopolymer paver blocks with and without the banana fiber with regard to compressive strength are assessed and contrasted with the conventional cement concrete paver blocks in accordance with Annex D of IS. 15658:2006^47^, and the values are tabulated in Table 6.

**Table 6:** Mechanical properties of geopolymer paver blocks.

Specimen type	Compressive strength (MPa)	Tensile strength (MPa)	Flexural strength (MPa)	Residual impact strength ratio	Water absorption (%)
CC	32	2.9	3.5	2.5	8.2
OGI35	41.6	4.7	6.8	2.7	5.5
F0.5OGI35	42.2	5.2	7.2	2.9	5.6
F1OGI35	43.8	5.4	7.6	3.2	5.7
F1.5OGI35	42.7	5.1	7.3	3.3	6.1

**Figure 4:**
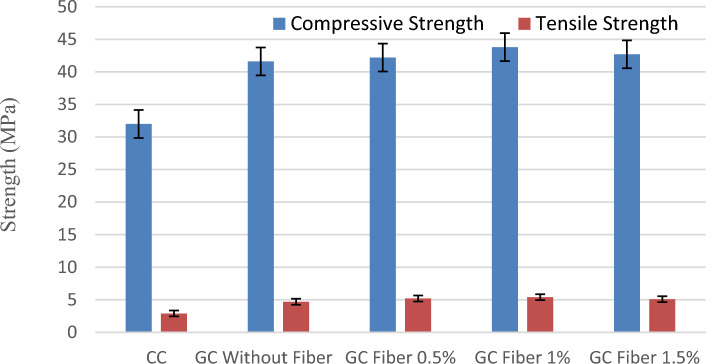
Variation of compressive and tensile strength of paver blocks.

Figure 4 compares the performance of cement concrete paver blocks and geopolymer paver blocks with and without the fibres.

From Fig. 4, it is pragmatic that the performance of the GC as paver blocks is superior to the conventional cement concrete specimens. Geopolymer concrete paver blocks exhibit about 30 percent higher compressive strength than conventional paver blocks. F1OGI35 specimens report that the presence of 1% banana fibre increases the compressive strength by about 5%. However, the addition of banana fibres of more than 1 percent leads to a reduction in workability and strength. The inclusion of fibres augments the structural integrity of the matrix, thereby enhancing the CS. Mostafa and Uddin^42^ reported a rise in CS through the employment of banana fibres in compressed earthen blocks. In the work, an optimized geopolymer paver block to withstand medium traffic conditions as prescribed by IS 15658:2006, which specified a requirement of 40 (MPa) compressive strength for geotechnical road material.

#### Tensile strength of paver blocks

The performance of the optimized geopolymer paver blocks with and without the banana fibre with regard to tensile strength are assessed and contrasted with the conventional cement concrete paver blocks as per Annex F of IS. 15658:2006^47^ and are listed in Table 6. Tensile strength performance is compared among paver blocks in Fig. 4. As of Fig. 4, it is inferred that geopolymer paver blocks yield about 62 percent higher tensile strength than cement paver blocks, and the presence of 1 percent fiber further increases the tensile strength of the paver blocks by about 14.8 percent. This increase in strength corresponds to the existence of high tensile and stiffness banana fibres in the concrete.

#### Flexural strength of paver blocks

**Figure 5:**
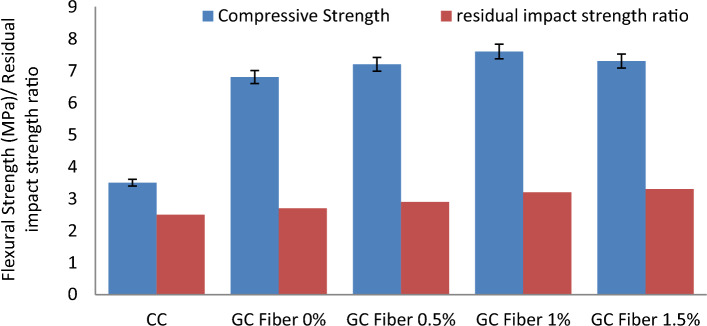
Variation of flexural strength and residual impact strength ratio of paver blocks.

The flexural strength of the geopolymer paver blocks with and without the presence of banana fiber is determined as per Annex G of IS. 15658:2006 and are tabulated in Table 6. Flexural strength performance is compared among paver blocks in Fig. 5.

Figure 5 reports that the flexural strength of the geopolymer specimens is about two times that of the cement paver blocks. The presence of fibres at 1% is found to enhance flexural strength by about 11.7 percent. The increment in strength owes to the capacity of the banana fibre to bridge the bending stress across them. After the inception of the crack at the yield load, these fibres bridge the concentrated load through them, thereby increasing the post-cracking behaviour of the paver block. This increases the load-bearing capability of the paver block. Elbehiryand Mostafa^68^ reported rise in flexural strength with the integration of banana fibres inside the cement concrete.

#### Impact resistance of paver blocks

The impact resistance of paver blocks with and without the presence of banana fibres in the iron ore slag-replaced specimens is assessed by conducting a drop weight impact test in accordance with the standards of ASTM D 2794. The resistance to impact is calculated by counting the quantity of blows needed to commence the first crack to the final crack by visual observation. The ratio between the corresponding absorbed energy at the failure to the first crack is termed residual impact strength ratio^22^.

The residual impact strength ratio of the paver blocks fabricated with the help of optimized banana fibre addition is compared with the results of paver without fibres and conventional specimens as calculated in Table 6. From the results, it can be inferred that the impact resistance of 1% banana fibre-reinforced paver blocks is improved to the maximum of 22% and 16% than the conventional and 0% banana fibres, respectively, as shown in Fig. 4. It is explicitly observed from the outcomes that the utilization of banana fibres acts as toughness reinforcing agents, which predominantly enhanced the mechanical properties, especially the impact resistance, in a wider manner in order to confirm the traffic load applications.

#### Water absorption (WA) capacity of paver blocks

WA Capacity of the paver blocks with and without the presence of banana fibre is determined as per Annex C of IS. 15658:2006 (IS:15658-2006) and are listed in Table 6.

From Table 6, it is seen that WA capacity of the GC paver blocks is lesser than cement concrete paver blocks. Utilization of banana fibres at 1% increases the absorption of water by about 3.6%s. This enhancement in water-absorbing capacity is because of the existence of fibres which have the tendency to absorb moisture. A similar type of increase in water absorption capacity has been observed in another research works as well^42^.

### Microstructural evaluation

**Figure 6:**
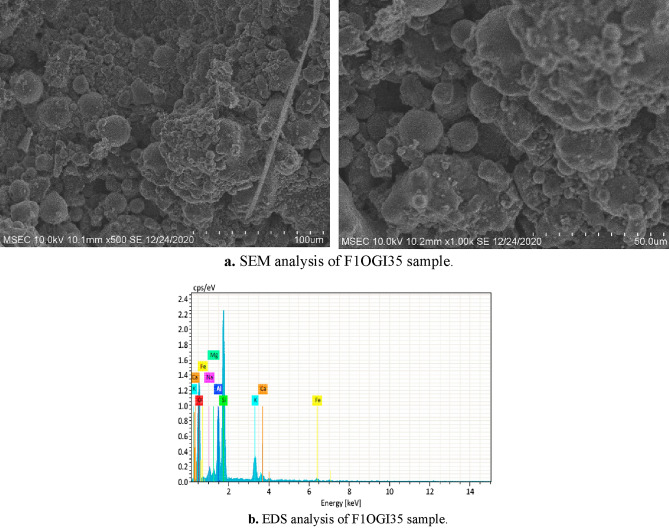
(**a**) SEM analysis of F1OGI35 sample. (**b**) DS analysis of F1OGI35 sample.

This investigation is carried out over the optimum geopolymer paver block sample F1OGI35 using Scanning Electron Microscopy (SEM) and Energy Dispersive Spectroscopy (EDS) and is portrayed in Fig. 6. SEM analysis is used to detect any flaws in the microstructure, and EDS analysis is used to find the chemical composition of the bond in the matrix.

Figure 6a implies that crystalline phases are explicit and are produced well. There is no trace of unreacted compounds, and this forms the base for the formation of superior bonds within the fibres and the matrix in the fly ash and slag-mixed fiber-reinforced GC samples. Utilization of iron slag as a substitute for M-sand has not involved or altered the geopolymerization reaction. This is evidence of the observed high strength of the geopolymer concrete samples. Figure 6b reports the presence of silica, aluminum and calcium. This substantiates the formation of the Al–O–Si bond and the Ca–S–H bond. Calcium-based compounds is due to the presence of GGBS in the matrix. Chindaprasirt et al.^66^ also confirmed that the increase in GGBS content leads to the formation of stable C–A–S–H or C–S–H gel thereby increasing the compressive strength.

## Analytical investigation through random forest

From the above experimental discussions, it is explicit that the expected output behavior compressive strength is solely dependent over the variable parameters discussed above, like molarity of the activator solution, % of GGBS utilization and % of partial substitution of river sand with iron slag. Hence developing a tangible relationship between the dependent variables and the output could save a lot of time, cost of materials and labor in this regard. The advent of machine learning (ML) has subsidized the pressure on the civil engineering domain as well. Prediction of the CS of concrete is proven technique by means of statistical models such as artificial neural network (ANN), K-nearest neighbor (KNN), partial least square regression (PLS), principal component regression (PCR) and random forest (RF)^69–72^. The ability of RF to respond near accurately for incomplete and noisy data to create indiscriminate results has made RF to be an extremely useful tool. RF algorithm’s ability to train the model from the instances in nature has made it to be one of the most reliable among the other renowned models^73^. RF is the most effective algorithm, which uses advanced ensemble techniques in predicting the target through multiple decision trees. The diverse applications of RF in various sectors include banking, health care, stock market, E-commerce and data analytics^74^. In this work key aim of this work is to build a random forest model to predict the compressive strength of GC for different parameters precisely and validate its performance through performance evaluators.

### Structure of random forest

**Figure 7:**
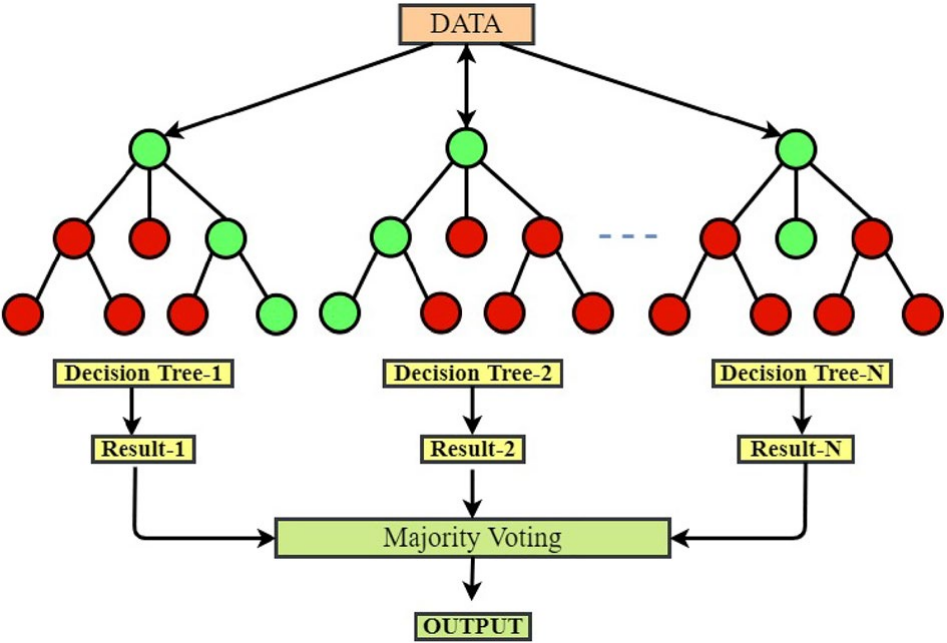
Structure of random forest algorithm.

The sample representation for the structure of RF algorithm to get a brief idea is represented in Fig. 7.RF is an advanced machine learning algorithm which considers multiple decision tree suggestions to develop an ear-accurate ensemble regressor/classifier outcome.

The over-fitting issues faced by decision trees are addressed by RF by improved accuracy of prediction, effective handling of datasets and development of model without hyper-parameter tuning. The key advantage of RF algorithm is that it employs a collective prediction technique which keeps the error low and deep decision trees.

### Performance evaluation parameters

In this study, the accuracy of the developed model and the predicted values can be ascertained using the performance evaluation parameters such as mean absolute error (MAE), normalized root mean squared error (NRMSE) and coefficient of determination (R^2^).

#### Mean absolute error (MAE)

Absolute error represents the error between the observed and the predicted value. Mean absolute error is defined as the ratio of the summation of absolute errors to the number of values. The relation is expressed in Eq. (1). MAE measures the error between the various observed and predicted values. The closeness of the value of MAE near zero represents the accuracy of the developed model.

1 $$\mathrm{MAE}=\frac{{\sum }_{\mathrm{i}=1}^{\mathrm{n}}|{\mathrm{sim}}_{\mathrm{i}}{-\mathrm{obs}}_{\mathrm{i}}|}{\mathrm{n}}$$

where MAE = mean absolute error, Sim = prediction value, obs = observed value, n = total number of data points.

#### Normalized root mean squared error (NRMSE)

Root mean squared error represents the variance between the observed values to the predicted values. The equation for RMSE is given in Eq. (2). Further normalized root mean squared error is defined as the ratio of root mean squared error to the difference between the ranges of observed values. NRMSE is just the normalization of RMSE of the observed variance in the model. The relation is expressed as Eq. (3). NRMSE is expressed in %. The value of NRMSE nearness to zero represents the accuracy of the developed model.

2 $$\mathrm{RMSE}=\sqrt{\frac{{\sum }_{\mathrm{i}=1}^{\mathrm{n}}({\mathrm{obs}}_{\mathrm{i}}-{\mathrm{sim}}_{\mathrm{i}}{)}^{2}}{\mathrm{n}}}$$

3 $$\mathrm{NRMSE}=\frac{\mathrm{RMSE}}{\mathrm{max}(\mathrm{obs})-\mathrm{min}(\mathrm{obs})}$$

where Max = maximum value of, Min = minimum value of.

#### Coefficient of determination (R^2^)

The coefficient of determination R^2^ represents the proportion of variance exhibited in the dependent variable with respect to the independent variable. It can simply be represented as a square of the correlation coefficient (r). The value of R^2^ ranges between 0 and 1, where the value near 1 represents the close association of datasets and the efficiency of the model. The R^2^ can be used as an indicator to determine the goodness of fit for the developed model.

4 $${R}^{2}=\frac{RSS}{SSS}$$

where RSS = regression sum of squares, SSS = summation of a sum of squares.

### Prediction and validation of random forest model

In the present study, 75% of the data set is used for training the model, and the other 25% is utilized for testing the precision of the developed model. The efficacy of the developed model can be ascertained from the performance evaluation parameters. Table 7 summarizes the range, effectiveness and observed value during the testing phase of the model.

**Table 7:** Performance evaluation parameters.

S.No	Parameter	Range	Effectiveness	Observed Value
1	Mean absolute error	–	**–**	0.35
2	Normalized root mean squared error	0–100%	Closeness to 0%more accurate	5.45
3	Coefficient of determination	0–1	Closeness to 1 more accurate	0.97

**Figure 8:**
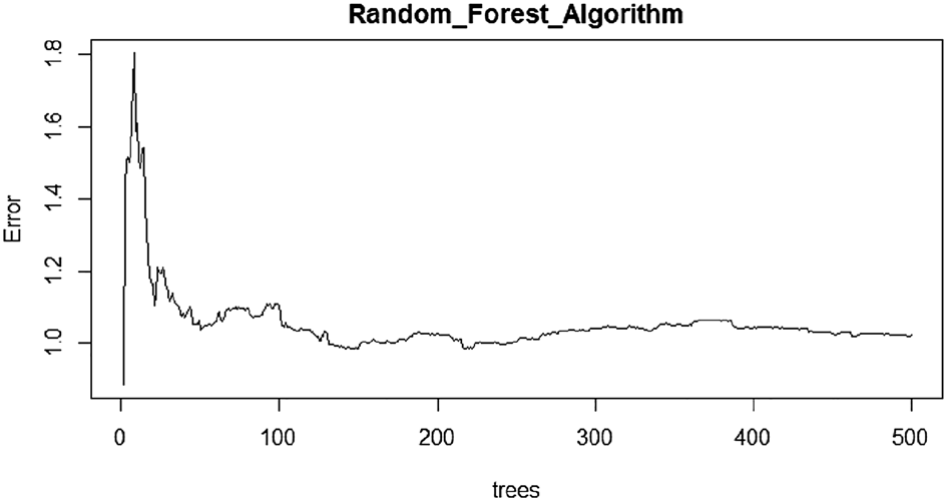
Optimization for number of decision trees in developed RF model.

From Table 7, it is explicit that the observed NRMSE and R^2^ values are very close 0 percent and 1, which claims the accuracy of the predicted model. Figure 8 depicts the iteration taken for the normalization of error by the developed RF model.

From the detailed discussions, it is clear that the developed model for the prediction of CS of the GC for input parameters such as molarity of the alkaline solution, % of GGBS utilization and % of partial substitution of river sand with iron slag.

## Conclusion

Extensive research work was performed to produce the high-impact strength paver blocks. Important parameters that affect the geopolymerization process, such as the molarity of sodium hydroxide solution and the fly ash-slag blend ratio, were optimized under heat curing. Utilization of iron slag as a partial substitute for river sand was determined. The final optimized geopolymer concrete is then applied as a paver bock with various proportions of the natural banana fibre and tested for its high impact strength that would sustain medium traffic conditions as per IS 15,658:2006. Findings from the test results and discussions could be abridged as follows.

- Geopolymer concrete exhibited enhancement in the CS with the increase in the molarity of the NaOH solution. 12 M of NaOH solution yielded the maximum rise in the CS.
- GC exhibited a raise in CS with the little incorporation of GGBS slag in the fly ash-based GC under heat curing. At an increased dosage of GGBS, a slight decrease in strength was observed. 20% partial substitution of fly ash with GGBS slag yielded maximum compressive strength.
- The incorporation of iron slag as a fine aggregate was determined to be beneficial in the Geopolymer concrete. 35 percent of the partial substitution of river sand with iron slag yielded the maximum compressive strength.
- Optimized Geopolymer concrete paver block with one percent banana fibre exhibited about a 5 percent rise in CS, 14.8 percent rise in tensile strength and 11.7 percent rise in flexural strength than the GPC paver block without the fibers.
- Also, it is noticed that the addition of 1% banana fiber in the ore slag optimized paver blocks enhanced the impact resistance by 22% more than the conventional cement concrete specimen and 15% than the pavers without fiber reinforcement.
- Geopolymer paver block with fiber content exhibited more water absorption capacity than the Geopolymer paver block without fiber. But this water absorption capacity is found to be less than the cement concrete paver blocks and is within permissible limits as prescribed by IS. 15658:2006.
- Fiber-reinforced geopolymer paver blocks synthesized in this study were determined to withstand medium traffic conditions as prescribed by IS. 15658:2006.
- The proposed RF model can precisely predict the twenty-eight days CS of GC for different parameters such as molarity of the activator solution, % of GGBS utilization and % of partial substitution of river sand with iron slag.
- The least values of NRMSE and nearness of R^2^ to 1 validate the effectiveness of the developed model in predicting the CS.

The works pave the path for the production of sustainable, eco-friendly paver blocks, and the hypothesis of the research as specified is achieved. This research work would open up the hefty potential of natural fiber-reinforced Geopolymer concrete as paver blocks to replace conventional cement paver blocks and reduce the strain over the environment by utilizing the indusrial btproducts as well. This research contributes an economical and eco-friendly paver block to society. The technique of utilizing a natural fibre to increase the impact strength and reduce brittleness has been proven effective, and further, its scope may be expanded to various applications.

## Data Availability

The datasets used and analyzed during the current study are available from the corresponding author on request.
